# Disruption of Monocyte and Macrophage Homeostasis in Periodontitis

**DOI:** 10.3389/fimmu.2020.00330

**Published:** 2020-02-26

**Authors:** Abdulrahman Almubarak, Kranthi Kiran Kishore Tanagala, Panos N. Papapanou, Evanthia Lalla, Fatemeh Momen-Heravi

**Affiliations:** ^1^Division of Periodontics, Section of Oral, Diagnostic and Rehabilitation Sciences, Columbia University College of Dental Medicine, New York, NY, United States; ^2^Herbert Irving Comprehensive Cancer Center, Columbia University Irving Medical Center, New York, NY, United States

**Keywords:** monocytes, macrophages, CD47, periodontitis, diabetes

## Abstract

Monocytes and macrophages are major cellular components of the innate immunity that play essential roles in tissue homeostasis. The contribution of different subsets of monocytes/macrophages to periodontal health and disease has not been fully elucidated. Type 2 diabetes mellitus (T2DM) is a risk factor for periodontitis. We hypothesized that the monocyte/macrophage signaling is perturbed in periodontitis-affected sites versus periodontally healthy sites and that this perturbation plays a critical role in the pathogenesis of periodontitis. Pairs of gingival tissue samples (each from a periodontally healthy and a periodontitis-affected site of the same patient) were harvested from 27 periodontitis patients, with and without T2DM. Each sample was processed to form a single-cell suspension, and a flow-cytometry panel was designed and validated to study monocyte and macrophage phenotypes. In separate experiments, the transcriptional changes associated with a pro-inflammatory phenotype were also examined in monocyte/macrophage subsets obtained from peripheral blood of patients with T2DM versus diabetes-free controls. A significantly higher proportion of intermediate (CD14^+^CD16^+^) monocytes was observed in periodontitis-affected tissues compared to healthy tissues. These monocytes overexpressed HLA-DR and PDL1 molecules, suggesting their activated inflammatory status. PDL1 increase was specific to intermediate monocytes. The ratio of M1/M2 macrophages was also significantly higher in periodontally affected sites, signifying an imbalance between inflammatory and repair mechanisms. We found a significantly higher expression of PDL1 in overall monocytes and M1 macrophages in periodontitis-affected sites compared to controls. Importantly, we identified a subpopulation of M1 macrophages present in periodontally affected tissues which expressed high levels of CD47, a glycoprotein of the immunoglobulin family that plays a critical role in self-recognition and impairment of phagocytosis. Analysis of the transcriptional landscape of monocytes/macrophages in gingival tissue of T2DM patients with periodontitis revealed a significant disruption in homeostasis toward a proinflammatory phenotype, elevation of pro-inflammatory transcription factors STAT1 and IRF1, and repression of anti-inflammatory JMJD3 in circulating monocytes. Taken together, our results demonstrate disruption of myeloid-derived cell homeostasis in periodontitis, with or without T2DM, and highlight a potentially significant role of these cell types in its pathogenesis. The impact of macrophage and monocyte signaling pathways on the pathobiology of periodontitis should be further evaluated.

## Introduction

Periodontitis is a chronic, multifactorial, inflammatory disease affecting the supporting structures of teeth. It is initiated by bacteria, can lead to tooth loss and contribute to systemic inflammation (1, 2). In recent years, it has been shown that the disease is a result of complex interactions between a dysbiotic bacterial biofilm and the host immune response that lead to a dysregulation of the inflammatory response and an imbalance between osteoblastic and osteoclastic activities (3, 4). Inability to resolve the pro-inflammatory milieu in periodontitis is believed to play a pivotal role in the pathogenesis of the disease (5). Of note, diabetes mellitus and cigarette smoking are two major risk factors for periodontal disease; they contribute to an exaggerated response to the bacterial challenge, leading to a higher prevalence and increased severity of periodontitis in affected individuals (6, 7).

Monocytes and macrophages are major cellular components of the innate immunity that play important roles in tissue homeostasis. Macrophages are the primary host defense elements against microbes and have the ability to migrate to the site of injury in a short period of time (8, 9). In recent years our understanding of monocyte/macrophage plasticity and the various cellular subtypes has significantly increased. Human monocytes are classified as classical, non-classical, or intermediate monocytes (10). Classical monocytes can become macrophages at the site of injury, and non-classical monocytes are mainly responsible for vasculature surveillance. Intermediate monocytes have a substantial role during the inflammatory cascade and are also known as hyperinflammatory monocytes (11–13). Monocyte life is short and non-classical monocytes live longer than classical monocytes (10). Non-classical monocytes accumulate at sites of chronic bacterial infection and produce low levels of pro-inflammatory, but high levels of anti-inflammatory cytokines (10).

Functional studies conducted in the pre-genomic era classified macrophages as pro-inflammatory macrophages (M1) and anti-inflammatory or resolution macrophages (M2) (14). M1 macrophages are associated with interleukin (IL)-12 and IL-8 and activate type 1 T helper (Th1) cells, while M2 macrophages are associated with transforming growth factor-beta (TGF-β), vascular endothelial growth factor (VEGF) or epidermal growth factor (EGF) and activate type 2 T helper (Th2) cells (15–18). During inflammation, macrophages arrive at the site of injury via diapedesis. This process is followed by M1 macrophage activation, which leads to the production of tumor necrosis factor (TNF)-α and IL-12 in the early stages of inflammation. In the resolution phase, M2 macrophages are activated along with TGF-β and IL-10 signaling. Additionally, M2 macrophages induce VEGF, which initiates the angiogenesis process. Activated TGF-β and platelet-derived growth factor (PDGF) signaling initiate matrix production and scar formation (19–21).

There is a sparsity of data regarding the frequency and phenotype of monocytes/macrophages in periodontal tissues. Additionally, there is a gap in the characterization and the delineation of function of monocytes/macrophages in human gingival tissue in periodontitis versus periodontal health. This study aimed to establish a protocol for single-cell analysis and precise characterization of human gingival tissue, and to explore the phenotypic changes of monocyte/macrophages in periodontitis compared to periodontal health in patients with and without T2DM.

## Materials and Methods

### Study Participants

The human subject protocol was approved by the Institutional Review Board at Columbia University Irving Medical Center, and informed consent was obtained from all study participants. Since periodontitis is site-specific and in order to avoid subject-based confounding factors, we studied pairs of gingival tissue samples (each from a periodontally healthy and a periodontitis-affected site of the same individual), harvested from 27 patients (6 with T2DM and 21 diabetes-free) undergoing periodontal surgery. Periodontitis-affected sites showed bleeding on probing (BoP), probing depth (PD) ≥ 5 mm, and clinical attachment loss (CAL) ≥ 3 mm; periodontally healthy (control) sites showed no BoP, PD ≤ 3 mm, and no CAL, as in previously published work (22). Higher levels of BoP, CAL, and PD are associated with a more severe periodontitis phenotype (1, 23). All patients were non-smokers. Patients with prediabetes, autoimmune diseases, and those who required antibiotic prophylaxis prior to dental appointments were excluded. In addition, peripheral blood mononuclear cells (PBMCs) from 6 diabetes-free individuals and 8 patients with T2DM were used for *ex vivo* experiments. Blood samples were obtained from the CALM laboratory at Columbia University Irving Medical Center and monocytes were isolated using a human CD16^+^ Monocyte Isolation Kit or a human CD14^+^ Monocyte Isolation Kit (Miltenyi Biotec, CA, United States) following the manufacturer’s instructions.

### Single-Cell Suspension Preparation

Gingival samples were processed to form a single-cell suspension, using a gentle MACS dissociator, a tissue dissociation kit and an optimized protocol. Briefly, gingival samples were kept in Dulbecco’s Phosphate-Buffered Saline (DPBS), washed twice with DPBS, and minced into small pieces. Each sample was then transferred into a c-tube which contained 2.35 mL of serum-free RPMI 1640 with 100 μL of Enzyme H, 50 μL of Enzyme R, and 12.5 μL of Enzyme A (Miltenyi Biotech, CA, United States). We used a Gentle mac-dissociator for mechanical and enzymatic disruption and kept the samples in a 37°C incubator for 30 min. The Gentle mac-dissociator was used three times and samples were placed in an incubator twice during the process. A 70-micrometer filter was used to achieve a single-cell suspension and each sample was washed with 15 ml RPMI solution and transferred into a centrifuge for 7 min under a 0.4 relative centrifugal field (RCF). The pellet was mixed with a freezing medium, which included 10% of dimethyl sulfoxide (DMSO) and 90% of fetal bovine serum (FBS). The samples were kept in an isopropanol chamber at −20°C for 24 h to preserve viability, then finally transferred into liquid nitrogen.

### Flow Cytometry

A flow cytometry panel was designed and validated to study monocyte and macrophage phenotypes as follows: CD14 (monocytes), CD16 (monocytes/macrophages), anti-human lineage cocktail CD3 (T cells), CD19 (B cells), CD20 (B cells), CD56 (dendritic cells), HLA/DR (monocytes/macrophages), CD163 (macrophages), CD206 (macrophages), MHC II (macrophages), CD47 (leukocyte surface antigen), CD45 (leukocyte surface antigen), CD80 (macrophages), CD 64 (macrophages), PDL1 (leukocyte surface antigen). Antibody-capture beads (CompBeads, BD Biosciences) were used as single-color compensation controls for each reagent used in the study. LSRII cytometer calibration was performed daily by use of rainbow fluorescent particles (BD Biosciences), after acquiring unstained and single-color control samples to calculate the compensation matrix.

### Quantitative Real-Time PCR

RNA was extracted using a Zymo kit (Zymo Research). OD ratios (260/280 and 260/230) were measured for determining RNA quality. cDNA was transcribed from l μg of total RNA, by use of the iScript RT kit (Bio-Rad). SYBR Green−based qRT−PCR was performed by use of a CFX Real-Time PCR Detection System (Bio-Rad). The levels of the target gene expression were quantified by the ΔΔ*CT* method using the ratio of the fold change in target gene expression versus the fold change in endogenous normalizer gene expression (18 s). The primer sequences are listed in Supplementary Table 1.

### Cell Culture and Luciferase Reporter Assay

Primary human monocytes/macrophages and THP1 monocytes were cultured in RPMI media containing 10% FBS at 37°C in a 5% CO_2_ and 1% penicillin/streptomycin (Gibco^®^, NY, United States). Primary human monocytes were differentiated to classically activated macrophages (M1) by addition of IFNγ (50 ng/ml) and lipopolysaccharide (LPS) from the Gram-negative periodontal pathogen *Porphyromonas gingivalis* (10 ng/ml). M2 (alternatively activated macrophage phenotype) was induced by addition of IL-4 (20 ng/ml) and IL-13 (24).

A renilla-luciferase reporter assay was carried out to examine the effect of NF-κB activation and inhibition on CD47 transcription. A total of 6 × 10^4^ cells was seeded on a 24-well plate in antibiotic-free media for 24 h. Cells were transfected with 12.5 ng renilla vector (Pierce) (used as an internal control) and 400 ng of pNF-κB-Luc and/or pRL−cytomegalovirus available through Stratagene (La Jolla, CA, United States). Following transfection, cells were harvested and lysed. Luciferase activity was tested using the Dual-Luciferase Reporter Assay System (Promega) as instructed by the manufacturer, and luciferase detection was measured on a Berthold Lumat LB9507 luminometer.

### Statistical Analysis

Means and standard deviations, or medians and ranges were calculated based on the distribution of data, and are used for data presentation. The Flow Cytometry Standard (FCS) files were analyzed using FCS Express 6 Flow software (Glendale, CA, United States). Non-parametric Wilcoxon matched-pair signed-rank tests, or parametric paired *t*-tests were used for statistical analysis, as indicated, based on the underlying distribution. Statistical significance was defined as *p* < 0.05.

## Results

**FIGURE 1:**
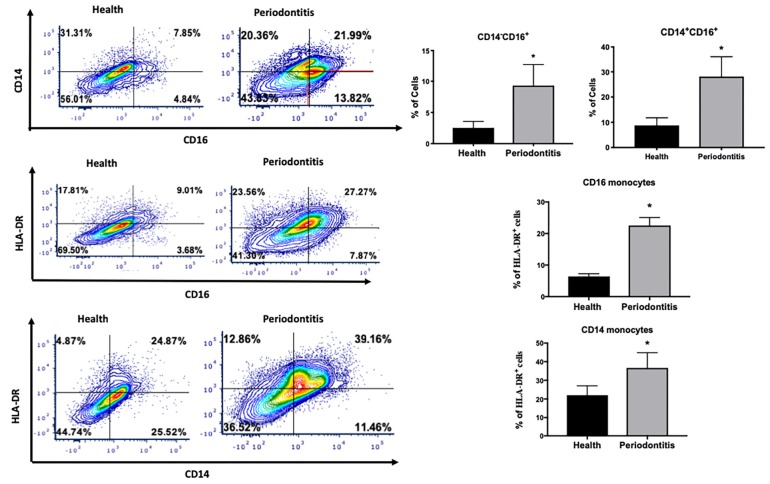
Single cell suspensions from matched periodontitis-affected and healthy gingival tissues were prepared and stained for UV Zombie, CD45, Lin (B220, CD19, Gr1, and Ter119), HLA-DR, CD16, CD14. **(A,B)** Representative counter plots of intermediate (CD14^+^CD16^+^) and non-classical monocytes (CD14^–^CD16^+^). Frequency of both cell types was higher in periodontitis-affected tissue compared to matched controls (*n* = 21; *p* < 0.05). **(C,D)** Representative counter plots and frequency of HLA-DR cells which are gated from live, CD45^+^Lin^–^CD16^+^ cells in periodontitis-affected gingival tissue and healthy gingival tissue (*n* = 21). Frequency of CD45^+^Lin^–^CD16 + HLA-DR^+^ cells was significantly higher in periodontitis-affected tissue compared to health (*p* < 0.05). **(E,F)** Representative counter plots (values indicate percentage of parent population) and frequency of HLA-DR cells which are gated from live, CD45^+^Lin^–^CD14^+^ cells in the periodontitis-affected gingival tissue and healthy gingival tissue (*n* = 21). Frequency of CD45^+^Lin^–^CD14^+^HLA-DR^+^ cells was significantly higher in periodontitis-affected tissue compared to health (*p* < 0.05). Data presented as mean ± SEM and *p* less than 0.05 was defined as significant (the symbol “*” indicates *p* < 0.05 compared to the healthy control).

We first assessed the distribution of monocyte subpopulations in periodontitis-affected sites in non-T2DM subjects. The gating strategy for monocytes is presented in Supplementary Figure 1. We identified a larger population of intermediate (CD14^+^CD16^+^) and non-classical (CD14^–^CD16^+^) monocytes in periodontitis-affected sites compared to healthy sites (*p* < 0.05) (Figures 1A,B). We also found significantly higher HLA-DR expression in both intermediate and non-classical monocytes in periodontitis-affected gingival tissues compared to healthy tissues, suggesting an activation and a pro-inflammatory profile of these monocytes (*p* < 0.05) (Figures 1C–F).

**FIGURE 2:**
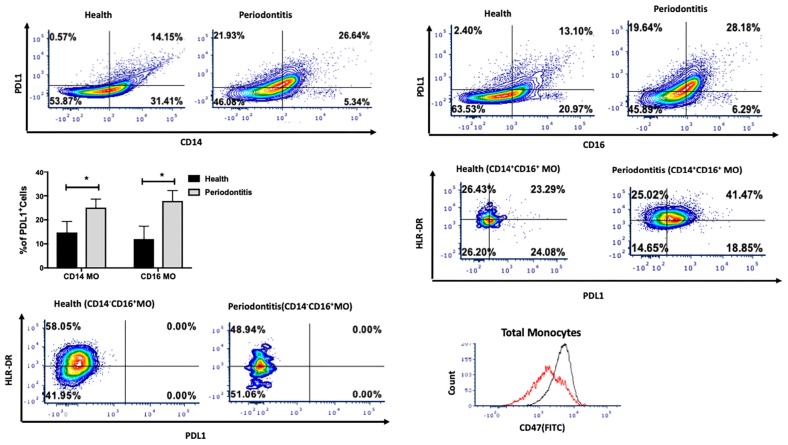
Single cell suspensions from matched periodontitis-affected and healthy gingival tissues were prepared and stained for UV Zombie, CD45, Lin, HLA-DR, CD16, CD14, and PDL1. **(A–C)**: Representative counter plots of CD14^+^PDL1^+^ and CD16^+^PDL1^+^ cells. Frequency of both cell types was higher in periodontitis-affected tissue compared to matched controls (*n* = 14; *p* < 0.05). **(D,E)**: Representative counter plots of intermediate and non-classical monocytes based on HLA-DR and PDL1 expression. HLA-DR^+^ PDL1^+^ population was only detectable in intermediate and not in non-classical monocytes. **(F)** Flow cytometry histogram of FITC-labeled CD47 expression in monocytes in periodontitis-affected tissue (black line) compared to healthy tissue (red line). The histogram was normalized to the number of events and depicts increase in CD47 in periodontitis-affected sites. Data presented as mean ± SEM and *p* less than 0.05 was defined as significant (the symbol “*” indicates *p* < 0.05).

The PD-1/PD-L1 pathway is one of the canonical pathways of immune evasion, tolerance, and T-cell function control. We next assessed the presence of PDL1 on monocytes. Interestingly, PDL1 expression was significantly higher in both intermediate and non-classical monocytes (Figures 2A–C), indicating the activation status of those monocytes in periodontitis-affected tissues (*p* < 0.05). Further characterization of intermediate and non-classical monocyte subpopulations showed a larger PDL1^high^HLA-DR^high^ population among intermediate monocytes but not among non-classical monocytes (Figures 2D,E). We also found higher CD47 expression on monocytes isolated from periodontitis-affected gingival tissues versus healthy tissues (Figure 2F).

**FIGURE 3:**
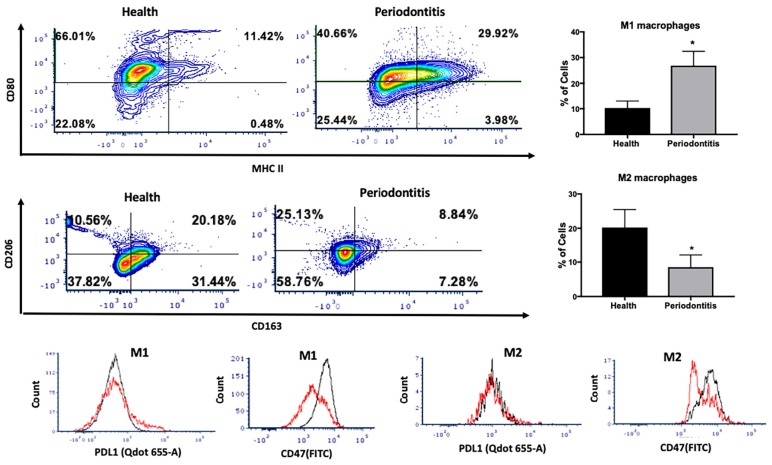
Single cell suspensions from matched periodontitis-affected and healthy gingival tissues were prepared and stained for UV Zombie, CD45, Lin, CD64, MHC II, CD80, CD163, CD206, PDL1, CD47. **(A,B)** Representative counter plots of M1 macrophages (CD80^+^MHC II^+^). Frequency of M1 macrophages was higher in periodontitis-affected tissue compared to controls (*n* = 21 pairs; *p* < 0.05). **(C,D)** Representative counter plots of M2 macrophages (CD206^+^CD163^+^). Frequency of M2 macrophages was lower in periodontitis-affected tissue compared to controls (*n* = 6 pairs; *p* < 0.05). **(E,F)** Flow cytometry histogram of CD47 (FITC) and PDL1 (Qdot 655-A) expression in M1 macrophages in periodontitis-affected tissue (red line) compared to the healthy tissue (black line) shows higher expression of both markers in periodontitis-affected sites. The histogram was normalized to the number of events. **(G,H)** Flow cytometry histogram of CD47 (FITC) and PDL1 (Qdot655-A) expression in M2 macrophages in periodontitis-affected tissue (black line) compared to the healthy tissue (red line) shows change in both markers in periodontitis affected sites in T2DM (the symbol “*” indicates *p* < 0.05 compared to the healthy control).

We then investigated the M1 and M2 macrophage phenotype in periodontitis-affected tissues in non-T2DM patients. The gating strategy for macrophages is presented in Supplementary Figure 2. M1 macrophages were significantly more abundant in periodontitis-affected tissues compared to healthy tissues (*p* < 0.05) (Figures 3A,B). Moreover, occurrence of M2 macrophages was significantly lower (*p* < 0.05) in periodontitis-affected tissues than in healthy tissues (Figures 3C,D). These findings are consistent with the notion that M1-mediated killing leads to collateral tissue damage which is cleared by healing-promoting M2 macrophages after elimination of the microbial invader (6).

CD47 is a glycoprotein of the immunoglobulin family involved in self-recognition and impairment of phagocytosis. The CD47 - signal regulatory protein α (SIRPα) axis plays a critical role in modulating macrophage function. CD47, a ‘self’ recognition marker expressed on several cell types, interacts with the immunoreceptor SIRPα expressed on macrophages to initiate inhibitory signaling that prevents macrophage phagocytosis of healthy host cells (24). Previous studies have suggested that cells may lose surface CD47 to enable phagocytic clearance (24). This perturbation may contribute to an impaired phagocytosis in periodontitis. Consistent with this notion, we found a significant over-expression of PDL1 and CD47 in M1 macrophages isolated from periodontitis-affected tissues compared to healthy tissues (*p* < 0.05) (Figures 3E,F). This higher expression was specific to M1 macrophages, and was not observed in M2-like macrophages (Figures 3G,H).

**FIGURE 4:**
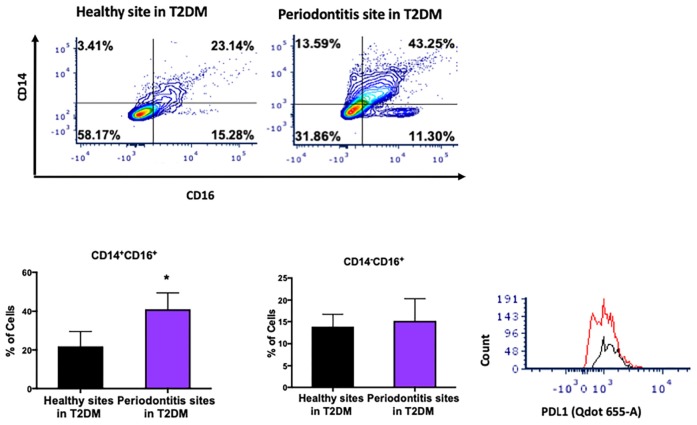
Single cell suspensions from matched periodontitis-affected and healthy gingival tissues harvested from patients with T2DM were prepared and stained for UV Zombie, CD45, Lin (B220, CD19, Gr1 and Ter119), HLA-DR, CD16, CD14. **(A–C)** Representative counter plots of intermediate (CD14^+^CD16^+^) and non-classical monocytes (CD14^–^CD16^+^). Frequency of intermediate monocytes was significantly higher in periodontitis-affected tissue compared to matched controls (*n* = 6; *p* < 0.05). **(D)** Flow cytometry histogram of CD47 (FITC) and PDL1 (Qdot655-A) expression in M2 macrophages in periodontitis-affected tissue (red line) compared to the healthy tissue (black line) shows higher PDL1 expression in periodontitis affected sites in T2DM (the symbol “*” indicates *p* < 0.05 compared to the healthy control).

T2DM is a major risk factor for periodontitis and disruption of monocyte/macrophage homeostasis has been implicated in the pathogenesis of both diseases. Using a similar approach, we investigated the distribution of monocyte subpopulations in periodontitis-affected sites in T2DM subjects and found a significant increase in the levels of intermediate monocytes (CD14^+^CD16^+^) in periodontitis-affected sites compared to periodontally healthy sites in patients with T2DM (Figures 4A,B). However, we did not find differences in the non-classical monocyte subpopulation (CD14^–^CD16^+^) (Figure 4C). Intermediate monocytes in periodontitis-affected sites also expressed high levels of PDL1 (Figure 4D).

**FIGURE 5:**
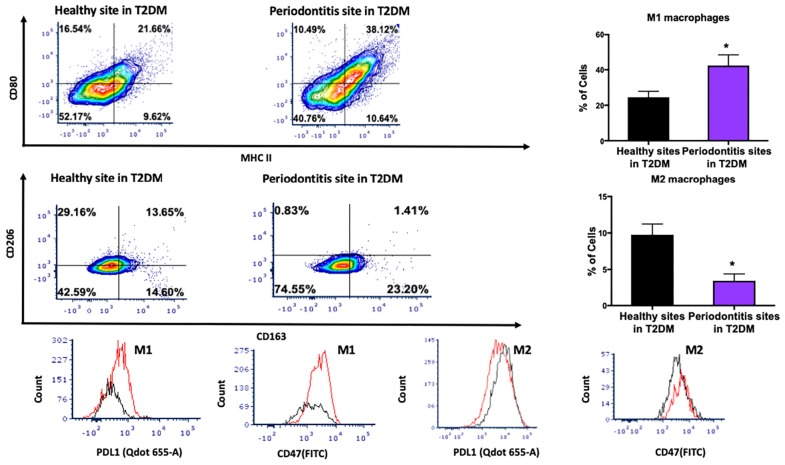
Single cell suspensions from matched periodontitis-affected and healthy gingival tissues from T2DM patients were prepared and stained for UV Zombie, CD45, Lin, CD64, MHC II, CD80, CD163, CD206, PDL1, CD47. **(A,B)** Representative counter plots of M1 macrophages (CD80^+^MHC II^+^). Frequency of M1 macrophages was higher in periodontitis-affected tissue compared to control tissue in T2DM (*n* = 6 pairs; *p* < 0.05). **(C,D)** Representative counter plots of M2 macrophages (CD206^+^CD163^+^). Frequency of M2 macrophages was lower in periodontitis-affected tissue compared to control tissue in T2DM (*n* = 6 pairs; *p* < 0.05). **(E,F)** Flow cytometry histogram of CD47 (FITC) and PDL1 (Qdot 655-A) expression in M1 macrophages in periodontitis-affected tissue (red line) compared to the healthy tissue (black line) shows higher expression of both markers in periodontitis-affected sites in T2DM. The histogram was normalized to the number of events. **(G,H)** Flow cytometry histogram of CD47 (FITC) and PDL1 (Qdot655-A) expression in M2 macrophages in periodontitis-affected tissue (red line) compared to the healthy tissue (black line) shows change in both markers in periodontitis affected sites in T2DM (the symbol “*” indicates *p* < 0.05 compared to the healthy control).

We then investigated the M1 and M2 macrophage phenotype in periodontitis-affected tissues in T2DM patients. M1 macrophages were significantly more abundant in periodontitis-affected tissues compared to healthy tissues (*p* < 0.05) in T2DM (Figures 5A,B). Additionally, occurrence of M2 macrophages was significantly lower in periodontitis-affected tissues compared to healthy tissues (Figures 5C,D). We found an increased expression of PDL1 and CD47 in periodontitis affected sites compared to healthy sites of patients with T2DM (*p* < 0.05) (Figures 5E–H).

Further, the percentage of intermediate monocytes was significantly increased in T2DM (21.86 ± 7.6 for healthy sites in T2DM patients versus 8.76 ± 2.99 healthy sites in non-diabetes individuals) as was the percentage of M1 polarized macrophages (24.52 ± 3.52 for healthy sites in T2DM patients versus 10.27 ± 2.70 healthy sites in non-diabetes individuals).

**FIGURE 6:**
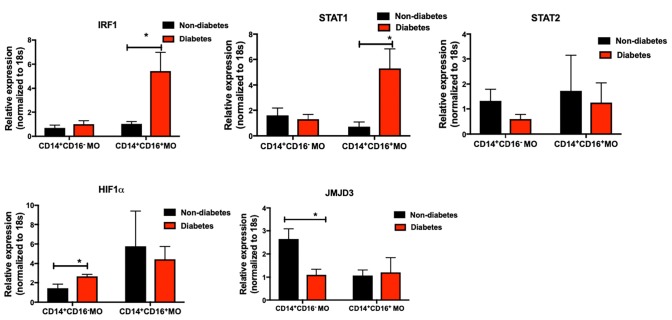
Primary human monocytes were isolated from the circulation of individuals with and without T2DM (at least 5 donors in each group). mRNA was isolated and qPCR was performed to analyze gene expression. **(A–E)** IRF1, STAT1, STAT2, HIF1α, JMJD3 levels were quantified by qPCR. 18 s was used as the endogenous normalizer. Data presented as mean ± SEM and p less than 0.05 was defined as significant (the symbol “*” indicates *p* < 0.05).

When periodontitis-affected sites were compared in patients with versus without T2DM, a diminished M2 polarization was noted (mean of 8.57 ± 3.5 for periodontitis-affected sites in non-diabetes individuals versus mean of 3.46 ± 2.91 for periodontitis-affected sites in T2DM). These findings suggested possible changes in the transcriptional landscape of monocytes/macrophages in T2DM. We therefore tested the transcriptional landscape of intermediate and non-classical monocytes isolated from patients with T2DM compared to non-diabetic controls. Interestingly, transcription factors associated with pro-inflammatory and anti-inflammatory phenotype were differentially expressed in circulating intermediate and classical monocytes in patients with T2DM (Figure 6). Specifically, IRF1 and STAT1 levels were significantly over-expressed in CD14^+^CD16^+^ T2DM monocytes (*p* < 0.05). STAT2 levels were similar in the different subpopulations. Expression of HIF1a was significantly higher in the diabetes CD14 monocytes (*p* < 0.05). In contrast, jumonji domain-containing protein D3 (JMJD3), an anti-inflammatory transcription factor for myeloid cells, was significantly under-expressed in diabetes CD14 monocytes (*p* < 0.05).

**FIGURE 7:**
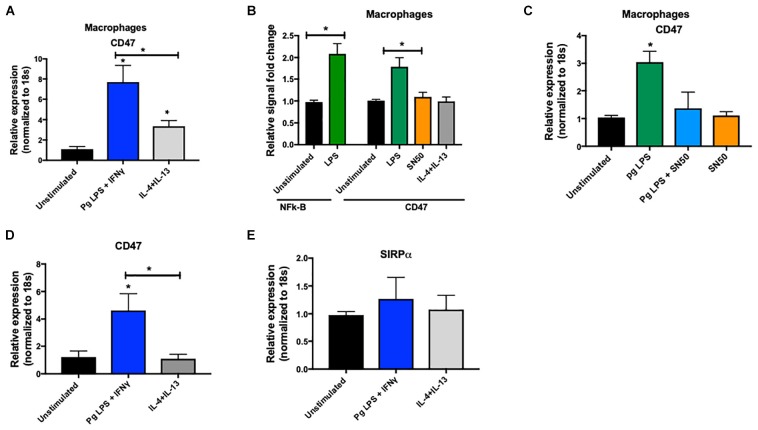
**(A)** THP1-derived macrophages were challenged with *P. gingivalis* LPS (10 ng/ml) and IFNγ (20 ng/ml) (M1 phenotype), or IL4 (40 g/ml) + IL13 (20 ng/ml) (M2 phenotype). Level of CD47 mRNA was quantified by qPCR after 24 h (at least 5 donors in each group). Data were normalized to 18 s. **(B)** Luciferase reporter assay was used to asses NF-κB and CD47 promoter activity in THP1-derived macrophages after challenge with *P. gingivalis* LPS or SN50 (20 μM). **(C)** THP1-derived macrophages were challenged with *P. gingivalis* LPS (20 ng/ml) and/or SN50 (20 μM) and level of CD47 mRNA was quantified by qPCR. Data were normalized to 18 s. **(D,E)** Primary human macrophages were challenged with *P. gingivalis* LPS (10 ng/ml) and IFNγ (20 ng/ml) (M1 phenotype), or IL4 (40 g/ml) + IL13 (20 ng/ml) (M2 phenotype). Levels of CD47 and SIPRα mRNA were quantified by qPCR after 24 h (at least 5 donors in each group). Data presented as mean ± SEM and p less than 0.05 was defined as significant (the symbol “*” depicts *p* < 0.05 compared to the unstimulated control, unless otherwise noted in the figure).

Finally, in separate experiments, we found a significantly higher expression of CD47 in classically activated macrophages (M1-like phenotype) compared to alternatively activated macrophages (M2-like phenotype) (Figure 7A). To examine whether CD47 was transcriptionally regulated, we assessed CD47 promoter activity in THP1-derived macrophages after stimulation with *P. gingivalis* LPS (a TLR4 agonist). The promoter of CD47 has a binding site for NF-κB, as previously described (25). Compared to unstimulated, control cells, we found that stimulation by *P. gingivalis* LPS activated NF-κB and higher transcription of CD47 (Figure 7B). Using SN50, a forty-one-residue synthetic peptide that contains a hydrophobic membrane-translocating region and prevents the nuclear localization sequence of NF-κB p50, we were able to repress the promoter activity of CD47 and decrease its mRNA transcription in the presence of LPS (Figure 7C). This finding demonstrates that the observed higher CD47 expression in macrophages is a result of gene activation at the transcriptional level. We validated these results in primary human macrophages. M1 polarized primary human macrophages showed a significant increase in CD47 expression as opposed to no change in the M2 polarized primary human macrophages. SIRPα expression did not show any significant differences in M1 versus M2 primary human macrophages (*p* < 0.05) (Figures 7D,E).

## Discussion

Periodontitis is a multifactorial, inflammatory disease which leads to loss of tooth-supporting tissues and contributes to the systemic inflammatory burden. The severity and extent of periodontitis depends on the interaction between triggering microbial factors and the host immune system (26) a process in which monocytes and macrophages play an essential role (4). Monocytes and macrophages are heterogeneous in nature and have the ability to exert different functions. In humans, three types of monocyte subsets are described based on relative expression levels of CD14 and CD16 surface proteins, namely classical, intermediate, and non-classical subsets. Transcriptomic and functional analyses of these subsets have revealed distinct functional properties. On the other hand, macrophages can be phenotypically classified into a M1 phenotype, which promotes the pro-inflammatory phase of the immune response, while M2 macrophages promote the healing and resolution phase (27). A perturbation of monocyte subtypes and M1 and M2 macrophage phenotypes has been described in other disease models.

In the present study, we have carefully characterized different subsets of monocytes and macrophages in periodontitis-affected tissues from patients with and without T2DM. Our analyses revealed a significantly higher abundance of intermediate monocytes (CD14^+^CD16^+^) as well as non-classical monocytes (CD14^+^CD16^++^) in periodontitis-affected tissues. Intermediate monocytes are the most pro-inflammatory subtype of monocytes, and their perturbation has been reported in a variety of inflammatory and autoimmune diseases (28, 29). Further, we observed higher expression in PDL1 and HLA-DR in both CD14 and CD16 monocytes. HLA-DR is an MHC class II cell surface receptor whose increased expression had been reported to be associated with autoimmune diseases and higher production of IL-1β and IFNγ upon Toll-like receptor stimulation (30). The co-stimulatory pathway consisting of the PD-1 receptor and its ligand, PD-L1, delivers inhibitory signals that regulate the balance among T-cell activation, tolerance, and immune-mediated tissue damage (31). In fact, as demonstrated in cancer biology, activation of this pathway contributes to T-cell exhaustion and lack of resolution during chronic infection (31). Interestingly, in our study we found high expression of PDL1 in intermediate monocyte subpopulation, but not in non-classical monocytes. Future mechanistic studies are needed to elucidate the role of PDL1-expressing intermediate monocytes in regulating the interplay between host defenses aimed at eradicating periodontal pathogens, and possible involvement of those cells in reduced T cell function and resolution in periodontal inflammation.

Our finding of a higher abundance in M1 macrophages and lower abundance in M2 macrophages in periodontitis-affected tissues may reflect a perturbation in tissue repair in periodontitis. Interestingly, we observed a higher abundance of M1 macrophage phenotype in healthy sites of patients with T2DM compared to healthy sites in non-diabetes patients. Macrophage-mediated regulation of chronic inflammation and especially M1 polarization plays a key role in the pathogenesis of diabetic complications (32, 33), and may explain the higher susceptibility of these individuals to periodontitis. These findings were corroborated by the *ex vivo* blood monocyte data indicating transcriptional changes in circulating blood monocytes of patients with T2DM versus non-diabetes individuals even before the cells reside in the gingival tissue. However, the present study provides information on the changes in monocyte/macrophages in gingival tissue of periodontitis susceptible individuals with and without T2DM, and the results should be interpreted in this context.

Intervention strategies aiming at an active shift from M1 to M2 phenotype could be a conceptually plausible therapeutic strategy in periodontitis, as suggested in a murine periodontitis model in which polarization of an M2 response was induced locally using C-C motif chemokine ligand 2 (CCL2) with controlled-release microparticles (34). CCL2 induces polarization of M2 macrophages at the injury site and activates anti-inflammatory cascades (35). The authors reported a decrease in M1 macrophages and less alveolar bone loss in the CCL2 group compared to the control group using micro-computed tomography. These data indicate that manipulation of the macrophage equilibrium could be a viable option in the treatment and/or prevention of periodontitis in susceptible individuals (34).

Interestingly, in patients with T2DM who are known to have a higher susceptibility to periodontitis, we observed a heterogenous reprogramming of circulating intermediate and non-classical monocytes toward M1 transcription activation, including an increase in STAT1, IRF1 in intermediate monocytes. A higher expression in HIF1α and a lower expression in JMJD3 in classical monocytes were also noted. Both IRF1 and STAT1 play critical roles in the host inflammatory response, M1 polarization, and T2DM pathogenesis (36, 37). HIF1α is both an anti-inflammatory transcription factor and one of the major mediators of impaired wound healing in diabetes (38). JMJD3, also called lysine-specific demethylase 6B (KDM6b), is an inducible histone demethylase which plays a role in macrophage polarization, function, and differentiation (39, 40) that induces M2 polarization via STAT6 signaling (41). Mechanistic studies have shown that JMJD3 also regulates IRF4, another key transcription factor that controls M2 macrophage polarization (40). IRF4^–/–^ macrophages display an M1 phenotype, reduced insulin signaling, and impaired glucose uptake by adipocytes (42). Since macrophages play a pivotal role in the pathogenesis of periodontitis in diabetes, macrophage function and phenotype modulation may be a reasonable therapeutic target for the treatment of periodontal inflammation and associated bone resorption in affected individuals (34, 43). Thus, it is important to explore mechanistic approaches to modulate this pathway in order to control the monocyte/macrophage phenotype in patients with T2DM with or without periodontitis.

In the current study, we found a higher CD47 expression in M1 macrophages of periodontitis-affected tissues. We also noted higher CD47 expression upon M1 polarization and *P. gingivalis* LPS challenge as opposed to no change in M2 polarized macrophages. Signal regulatory protein alpha (SIRPα) and CD47 have an important role during cancer progression (33) and blocking CD47-SIRPα interaction has been shown to promote the destruction of cancer cells by phagocytes, macrophages, and neutrophils (33). Blocking the interaction between SIRPα and CD47 is also important for cell migration and phagocytosis (34). This finding can explain the impaired phagocytosis phenotype that has been reported in periodontal disease (35). Also, it has been confirmed that a reduced concentration of SIRPα or CD47 will affect osteoclasts (36). As CD47 has a binding site in its promotor for NF-κB, we showed promotor activity of CD47 after *P. gingivalis* LPS stimulation and NF-κB activation.

In a recent study on monocyte/macrophage trajectory, an increase in the M1 phenotype was found in the intermediate monocyte population and the non-classical monocyte population in an aging cohort, supporting the hypothesis of a systemic pro-inflammatory polarization of monocytes in older patients (44). These results can explain our findings of a concurrent higher abundance in M1 macrophages, intermediate monocytes, and non-classical monocytes. Consistent with our results, an increase in the percentage of inflammatory monocytes and M1 macrophages has been reported in the circulation of patients with T2DM (45, 46). The dynamic changes of monocyte/macrophage and myeloid cell trajectories should be evaluated in future mechanistic studies (47). Similarly, the role of memory macrophages in the periodontal tissues, is poorly understood. While it has been shown that certain subsets of macrophages are maintained by circulating monocytes, other subsets have been reported to operate independently (27, 47). Moreover, the distribution and role of those macrophages, the mechanisms of recruitment of intermediate monocytes to the inflammatory niche, and their ability to differentiate into macrophages are still unclear in the context of periodontitis and need to be investigated in future studies. Specifically, functional phagocytosis analysis and precision reprogramming of the macrophages in response to the environment and the bacterial challenge in the gingival milieu should also be evaluated in future studies.

## Conclusion/Future Directions

Our results demonstrate for the first time a disruption of myeloid-derived cell homeostasis in periodontitis and highlight a potentially significant role of these cell types in its pathogenesis. Our findings set the stage for future studies that will further dissect the role of monocyte/macrophage signaling pathways in the pathobiology of periodontitis and the therapeutic potential of M2 macrophage induction in the resolution of inflammation.

## Data Availability Statement

The datasets generated for this study are available on request to the corresponding author.

## Ethics Statement

The studies involving human participants were reviewed and approved by the Columbia University Medical Center. The patients/participants provided their written informed consent to participate in this study.

## Author Contributions

AA performed experiments and drafted the manuscript. KT performed experiments. PP interpreted the data and revised the manuscript. EL conceived the idea, interpreted the data, and revised the manuscript. FM-H conceived the idea, performed experiments, analyzed and interpreted the data, contributed reagents, materials, analysis tools, and revised the manuscript.

## Conflict of Interest

The authors declare that the research was conducted in the absence of any commercial or financial relationships that could be construed as a potential conflict of interest.
