# Supplementary figures and images for: Disruption of Monocyte and Macrophage Homeostasis in Periodontitis
Source: Front Immunol. 2020 Feb 26;11:330. doi: 10.3389/fimmu.2020.00330 (PMC7067288; doi:10.3389/fimmu.2020.00330)

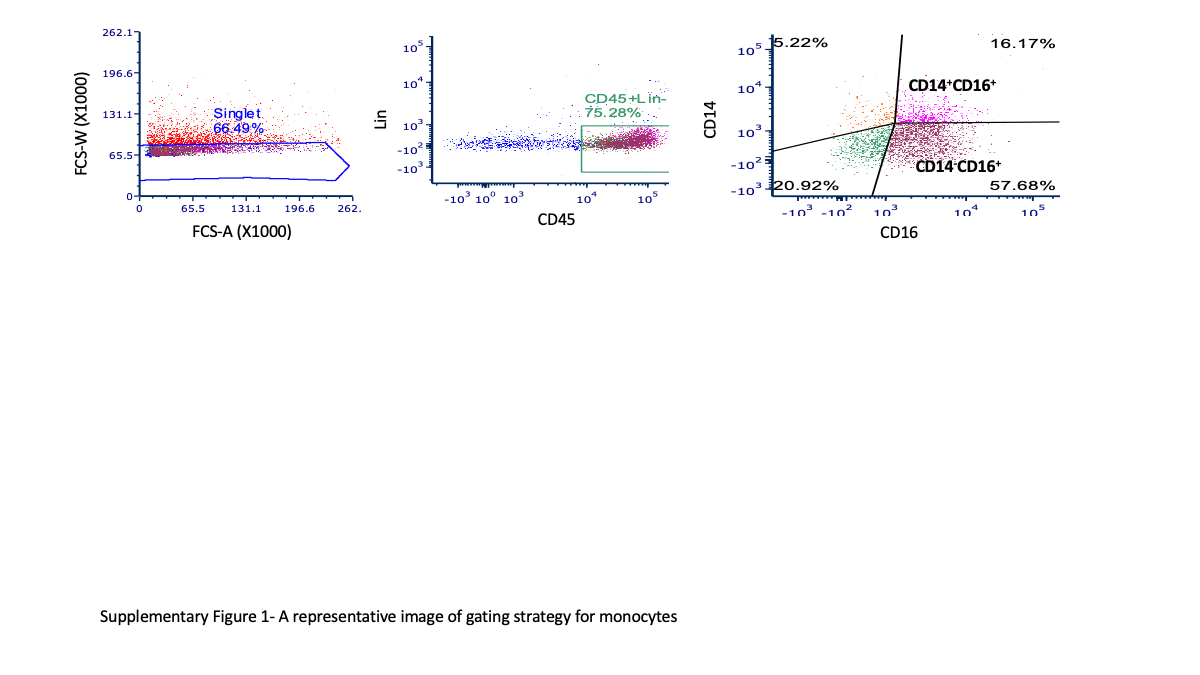

Supplement: Supplementary file 1 [file Image_1.tiff]

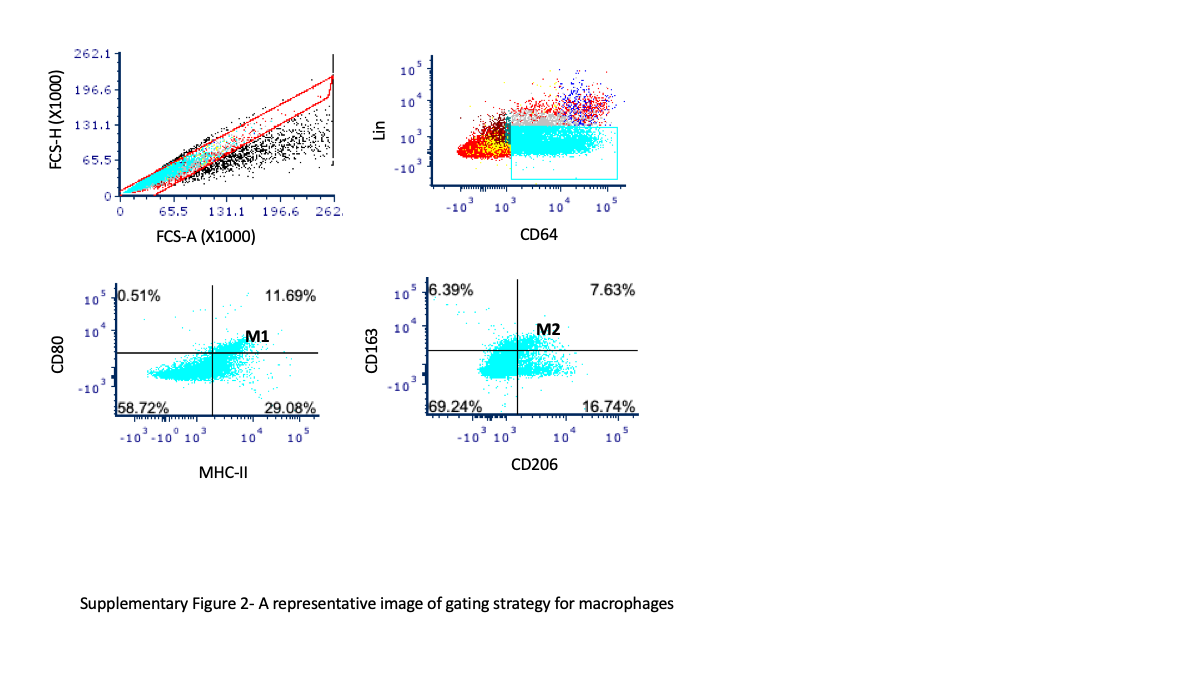

Supplement: Supplementary file 2 [file Image_2.tiff]
