# Supplementary material for: Disruption of Monocyte and Macrophage Homeostasis in Periodontitis
Source: Front Immunol. 2020 Feb 26;11:330. doi: 10.3389/fimmu.2020.00330 (PMC7067288; doi:10.3389/fimmu.2020.00330)
Supplement: Supplementary file 3 [file Table_1.doc]

**Supplementary Table 1-** PCR primer sequences used for the present study

| **Gene** | **Forward sequence** | **Reverse sequence** |
| --- | --- | --- |
| **CD47** | GGCAATGACGAAGGAGGTTA | ATCCGGTGGTATGGATGAGA |
| **IRF1** | GAGGAGGTGAAAGACCAGAGCA | TAGCATCTCGGCTGGACTTCGA |
| **JMJD3** | GCACCCCAGCAAACCATATTA | GTGCTCTGACTCGTACAGTTG |
| **STAT1** | GGAACTTGATGGCCCTAAAGGA | ACAGAGCCCACTATCCGAGACA |
| **STAT2** | CTGAACTATGAGTGTGGCCG | ACAGGTGTTTCGAGAACTGG |
| **HIF1α** | GAAAGCGCAAGTCTTCAAAG | TGGGTAGGAGATGGAGATGC |
| **SIRPα** | TCGAGTGATCAAGGGAGCA | CCTGGACACTAGCATACTCTGAG |
| **18S** | CTACCACATCCAAGGAAGCA | TTTTTCGTCACTACCTCCCCG |
